# Reduced Stocking Density and Provision of Straw in a Rack Improve Pig Welfare on Commercial Fattening Farms

**DOI:** 10.3389/fvets.2021.656211

**Published:** 2021-12-08

**Authors:** Katharina Schodl, Lisa Wiesauer, Christoph Winckler, Christine Leeb

**Affiliations:** ^1^Division of Livestock Sciences, Department of Sustainable Agricultural Systems, University of Natural Resources and Life Sciences, Vienna, Austria; ^2^Doctoral School of Sustainable Development (dokNE), University of Natural Resources and Life Sciences, Vienna, Austria

**Keywords:** animal welfare, fattening pigs, enrichment, space allowance, animal-based indicators, on-farm research

## Abstract

The aim of this study was to investigate the impact of reduced stocking density in combination with provision of additional enrichment material on pig welfare on-farm. On three growing-finishing farms in Austria, in total 974, 413, and 70 pigs were kept at 1 m^2^/pig and straw or hay in a rack as additional enrichment material (improved pens, IP; *n* = 6–38 pens per farm) or in pens complying with the minimum legal requirements of 0.7 m^2^/pig (control pens, CP; *n* = 6–37 pens per farm). Behavior as well as skin, tail and ear lesions and pig soiling were assessed at the beginning and in the middle of the fattening period, while performance data were recorded at the end of the fattening period. Data analysis was performed for each farm separately using generalized linear and linear mixed models or Mann-Whitney U-Test. Pigs in IP less frequently manipulated pen mates' ears (Farm 1: $P_{treatment^{*}assessment}$ = *0.002*; Farm 2: *P* = *0.002*) and body (Farm 1: *P* = *0.021;* Farm 2: *P* = *0.015*) than in CP. Prevalence of skin, tail and ear lesions and soiled pigs did not differ between treatments. In only one farm, average daily weight gain was higher in IP than in CP (Farm 1: *P* = *0.003*). Our findings indicate that increased space allowance and provision of substrate can improve aspects of animal welfare within existing pig fattening systems, without requiring irreversible constructional modifications to the system.

## Introduction

For labor and cost efficiency reasons, in most European countries fattening pigs are kept indoors under controlled climatic conditions with high stocking densities and fully slatted floors. When well-managed, clinical health can be maintained, whilst other aspects of animal welfare, especially the expression of highly motivated normal behaviors, are often not met. Low space allowance and lack of structured pen design do not allow pigs to separate lying and dunging areas, limit access to resources such as food and water and decrease the possibility to avoid other pigs (1–3). The barren environment together with a lack of exploratory material promotes the development of behavioral disorders such as tail and ear biting with detrimental effects on animal welfare and economic performance (4, 5). Tail and ear biting are multifactorial behavioral disorders caused by numerous internal (e.g., genetic predisposition, sex) and external (e.g., group size, floor type, feeder space, temperature) risk factors, which makes control and prevention demanding (4, 6).

In light of the increased public concern about animal welfare in intensive pig fattening units and the implementation of the EU Council Directive 2008/120/EC, effective measures to improve animal welfare are needed, which may be readily implemented in existing commercial fattening farms. Although EU legislation provides a detailed description of characteristics of adequate enrichment material for pigs, implementation on farms may still be inadequate in terms of presentation, location or quantity or even not complying to regulations at all (7). Studies on environmental enrichment for pigs found that straw presents the most suitable enrichment material in pigs (8) and that pigs interact often and persistently when provided loosely in racks (9). In Sweden, where rearing of pigs with intact tails is mandatory, the minimum space allowance at which the risk of tail biting and other detrimental behaviors is assumed to be reduced is calculated using the formula 0.17 + (live weight kg)/130 (10). According to this formula, pigs with a live weight of 110 kg should be provided a space allowance of ~1 m^2^ per animal, whereas Austrian legislation demands a minimum space allowance of 0.7 m^2^ per pig of up to 110 kg live weight (11).

Therefore, the aim of this study was to investigate, whether a reduced stocking density resulting in a higher space allowance (1 m^2^ instead of 0.7 m^2^ per finishing pig) in combination with provision of straw or hay in a rack as additional enrichment material improves welfare of growing-finishing pigs. Following the implementation of these measures, we expected an increase in exploration of the enrichment material and therefore a reduction of detrimental behaviors such as manipulation of pen-mates. Furthermore, prevalence of skin, ear and tail lesions was expected to decrease and growth rate of pigs to improve whereas the risk of soiling of pigs was expected to increase.

While there is a considerable body of evidence on the effects of stocking density and environmental enrichment on pig welfare [see review by (7) or original papers such as (8–10)], most of the studies have been conducted in an experimental setting and therefore may have limited applicability to commercial farms. Furthermore, studies involving multiple commercial farms either are cross-sectional and do not involve interventions [e.g., (12, 13)] or involve only one farm and thus do not allow assessing variability of intervention outcomes [e.g., (14)]. Peer-reviewed studies investigating a combination of improvement measures on more than one commercial farm under “real management conditions” are largely missing. Thus, by using a multi-farm approach, we furthermore aimed at exploring the effects of these measures under varying “on-farm” conditions.

## Animals, Materials and Methods

### Study Design

The study was carried out as an intervention study on three commercial pig fattening farms in Austria between May 2013 and February 2014. The in total 1,457 pigs involved in the study (see Table 1) were not subject to any procedures other than standard procedures on conventional pig fattening farms such as tail docking (on two farms). Since all assessments were carried out visually and the implementation of a higher space allowance and straw as environmental enrichment are generally regarded beneficial for animal welfare, no ethical approval was necessary for this study.

**Table 1:** Details of intervention measures implemented on participating farms (CP, control pens; IP, improved pens).

	**Farm 1**		**Farm 2**		**Farm 3**	
	**CP**	**IP**	**CP**	**IP**	**CP**	**IP**
Space allowance	0.76 m^2^/pig	1.03 m^2^/pig	0.75 m^2^/pig	1.06 m^2^/pig	0.71 m^2^/pig	1.11 m^2^/pig
Material in rack		Straw		Straw		Hay
		42.5 g/pig/day		144 g/pig/day		267 g/pig/day
Replications	5	5	5	5	6	6
Pens	37	38	24	12	6	6
Pigs/pen	14–16	10–13	10–13	7–10	8–9	5–6
Total number of pigs	556	418	246	167	42	28

All pens contained mixed sex groups of female and castrated male crossbreed pigs (F1 Large White^*^Landrace sows sired by a Pietrain boar) which were ~12 weeks old at the beginning of the study. Pigs in control pens (CP) were kept at 0.7 m^2^ per animal according to Austrian legal minimum requirements (11). In improved pens (IP) the number of animals per pen was reduced, resulting in a target space allowance of 1 m^2^ per pig (from 85 to 110 kg live weight). Consequently, the number of pigs per trough and per drinker was reduced, ranging from eight to 16 animals and five to 13 animals per drinker in CP and IP, respectively. Length of troughs corresponded to Austrian legal minimum requirements, providing 33 cm feeding space per animal in CP (11), whereas feeding space increased to 40.6–52.8 cm per pig in IP. Depending on the availability on the farms, straw or hay was provided daily in a rack (with 2 cm between bars) installed above the feeding trough. All pens were equipped with fully slatted floors, forced ventilation and heating. Farm 2 and Farm 3 provided the enrichment material in racks already to weaners in the rearing pens. All pens additionally contained a wooden block on a chain or a chain with plastic disks (some pens on Farm 2) as standard enrichment object. On Farm 1 in total 11 pens (six IP, five CP) did not contain this standard enrichment object at the beginning of the fattening period and on Farm 2 this was the case in one pen (IP) in the middle of the fattening period. Pigs were fed a GMO-free standard diet using a liquid feeding system on all three farms.

Initially, it was planned to also include omission of tail docking in the IP treatment to investigate the potential of this combination of measures for fattening pigs with intact tails in compliance with EU Council Directive 2008/120/EC (15). However, tail docking status of pigs varied between the three farms due to different management situations. All pigs on Farm 1 were tail docked because the associated rearing farm was concerned about tail biting in the rearing phase and therefore refused to omit tail docking. On Farm 3, all pigs had intact tails due to routine omission of tail docking already prior to the project, whilst on Farm 2 IP pigs had intact tails and pigs in CP were tail docked. At a live weight of approximately 30 kg, pigs were assigned randomly to CP or IP (in Farm 2 random allocation within the animals of same tail docking status). Details on the implementation of the intervention measures on participating farms are provided in Table 1.

### Data Collection

Behavior and physical appearance were recorded twice on-farm (assessment I and II). Assessment I was carried out on the day the pigs were transferred to the fattening pens and served as baseline information for physical appearance data, e.g., tail- and ear lesions. Assessment II was performed in the middle of the fattening period (at a ~70 kg live weight).

#### Behavioral Observations

Behavioral observations took place in three out of five replications on Farm 1, four out of five replications on Farm 2 and four out of six replications on Farm 3. All behavioral observations were performed by the same observer. For both assessments I and II, observation of the behaviors specified in Table 2 was carried out at pen level using direct continuous observation. After a 2 min waiting period, which allowed the pigs to get familiar with the presence of the observer, each pen was observed for 10 min. Behavior was recorded as number of events occurring in the pen. If a pig interrupted a behavior for longer than 10 seconds this was recorded as a new incidence. The time at which observations took place varied between farms (8:30 am−2:15 pm, 8:40 am−4:00 pm, 12:30 pm−3:10 pm on Farm 1, 2, 3, respectively) according to farm management schedules.

**Table 2:** Ethogram for continuous behavioral observations.

**Behavior**	**Description**
Manipulation of enrichment object	Touching, sniffing, licking or biting the object [usually a wooden block on a chain or a chain with plastics disks [modified after (16, 17)]
Manipulation of straw/hay in a rack	Rooting, sniffing, chewing or manipulating straw/hay with direct contact with the rack and/or the material [modified after (16, 17)]
Manipulation of body of pen mates (other than tails and ears)	Touching, sniffing, rooting, licking, biting and chewing another pig's shoulder/flank, hindquarters, limbs, anogenital or belly area (16, 18)
Tail biting	Manipulating, sucking or chewing another pig's tail or taking the tail into the mouth without obvious chewing or sucking motion (“tail-in-mouth-behavior”) (19, 20)
Ear biting	Manipulating, sucking, chewing or taking another pig's ear into the mouth (19)
Head knocks	A rapid thrust upwards or sideways with the head against any part of the body of a pen mate (21)
Fighting	Forceful pushing of a pen mate with or without biting (16)

#### Lesions and Soiling of Pigs

Assessment of lesions on skin, ears and tails as well as soiling of pigs was based on definitions from scientific literature and previous on-farm studies (Table 3). From inside the pen, all pigs were assessed and the number of animals affected was recorded for all indicators at pen level. Scoring was carried out by two trained observers. Based on assessments of 30 pens on one farm on 1 day, inter-observer agreement at pen level (numbers of animals affected, allowing a deviation of one animal between the observer counts) was acceptable (r_s_ ≥ 0.7) for all indicators of physical appearance.

**Table 3:** Description of assessment of lesions and soiling of pigs.

**Indicator**	**Description**
Soiling of pigs	> 20% of the body surface soiled with feces (one side of the pig assessed) (22)
Lesions on the flank/hindquarters	Round superficial skin lesion >1 cm in diameter (skin broken; both sides of the pig assessed)
Scratches in flank/shoulder region	Linear superficial skin lesions > 3 cm (one side of the pig assessed)
Scratches on hindquarters	Linear superficial skin lesions > 3 cm (one side of the pig assessed)
Ear tip lesions (point where medial and lateral edges of the pinna meet)	Score 1—wound/scab or necrotic tissue starting from the tip; >1 cm in diameter [modified after (23)]
	Score 2—obvious tissue loss
Ear edge lesions (area between ear lobe and tip)	Score 1—wound/scab or necrotic tissue >5 cm length [modified after (23)]
	Score 2—obvious tissue loss
Tail lesions [modified after (24)]	Score 1—superficial lesion (fresh blood or scab) on tail tip, no signs of infection
	Score 2—more severe bleeding or obvious scab, first signs of infection (swelling, reddening)
	Score 3—severe damage of tail, deeper tissues is visible, obvious infection (swelling, reddening, purulence)

#### Performance Data and Treatments

Per fattening pen, pigs were weighed as a group at the beginning and end of the fattening period before transport to the abattoir. Number of fattening days was obtained on pen level from feeding computers. Average daily weight gain (ADG) was calculated from the difference between the average initial and final weight divided by the number of fattening days. Lean meat content was obtained from the abattoir's classification protocols at animal level. Per batch, farmers recorded veterinary treatments and mortality for each pen.

### Data Analysis

The farms varied markedly in size, structure and management and this variability was an important aspect of this study with regard to the implementation of improvement measures. Statistical analyses were performed for each farm separately because rather than investigating the average change in animal welfare indicators on all three farms by adding a farm effect we were interested in comparing how animal welfare was affected on each farm and if the direction of the change was consistent. Pen was the statistical unit for behavioral data as well as indicators of physical appearance. On Farm 1 and Farm 2, performance data were analyzed at the level of pairs of adjacent pens, which shared one feeding trough, while on Farm 3, analysis took place at the pen level (one trough per pen).

#### Statistical Analyses

Statistical analyses were performed using the SAS software package, Version 9.4 for Windows (25). Behavioral and physical appearance data were analyzed using a generalized linear mixed model (PROC GLIMMIX) with assessment (I, II), treatment (CP, IP) and the interaction term as fixed effects. Replication (batch) was included as random effect and pen was included as the repeated measures unit. Due to convergence issues, head knocks and fighting were merged to ‘agonistic behavior’ as were the different lesion scores for ear tip, ear edge and tail to total lesions per region. The sample size on Farm 3 was too small for the hierarchical structure of the data and the model did not converge for most of the indicators (26). Hence, differences between treatments on Farm 3 were examined for each assessment using a Mann-Whitney U-Test (PROC NPAR1WAY).

Prior to analysis of lesions on skin, ears and tails as well as soiling of pigs, correlations between the individual indicators were assessed (PROC CORR option SPEARMAN). Based on these results, the indicator ‘scratches on hindquarters’ was excluded from the analysis because it correlated with ‘scratches flank’ (r_s_ = 0.73 for Farm 1 and r_s_ = 0.54 for Farm 2 and Farm 3).

Performance data were analyzed using a mixed model (PROC MIXED) with treatment (CP, IP) as fixed and replication as random effect. Data on lean meat content were recorded for each animal individually at the abattoir and therefore the random effect was defined as feeding valve (feeding trough shared by two adjacent pens) nested in replication. Initial body weight was retained in the model as covariate if it differed significantly between treatments (PROC MIXED). Residuals were checked graphically for normal distribution using Q-Q-plots. To avoid spurious findings we controlled for false discovery rate according to the procedure suggested by Benjamini and Hochberg (27). Thus, results were considered significant for *P* < 0.037, *P* < 0.037 and *P* < 0.048 for Farm 1, Farm 2 and Farm 3, respectively.

## Results

Results for the behavioral observations are presented in Tables 4–6.

**Table 4:** Frequency of behaviors (number of events/100 animals^*^10 min; LS-means ± standard error of means) for pigs in IP (improved pens) and CP (control pens) during assessments I (baseline) and II (middle of the fattening period) on Farm 1.

**Farm 1**	**Assessment I**		**Assessment II**		***P***		
	**CP**	**IP**	**CP**	**IP**	**T**	**A**	T^*^A
Manipulation objects	5.6 ± 4.7	13.1 ± 9.0	14.5 ± 4.5	28.6 ± 8.1	0.189	0.093	0.866
Manipulation straw/hay	–	54.4 ± 10.3	–	66.3 ± 9.1	–	0.236	–
Manipulation pen mates^a^	9.5 ± 3.7	18.6 ± 5.8	121^b^ ± 31.7	72.4^b^ ± 20.2			**0.021**
Tail biting	0.6 ± 0.4	0.8 ± 0.6	11.0 ± 2.1	4.5 ± 1.5	0.581	**<0.001**	0.259
Ear biting	9.3 ± 2.6	11.7 ± 3.3	40.5^b^ ± 6.0	10.5^b^ ± 2.8			**0.002**
Agonistic behavior	5.4 ± 2.0	1.6 ± 1.2	14.0 ± 3.1	4.5 ± 1.8	**0.007**	0.045	0.948

P-values are given for the effects of treatment (T), assessment (A) and the interaction term (T^*^A) and are considered significant at P < 0.037 (bold values). –, effect not included in the model.

a *Absence of enrichment object was significant and thus retained in the model*.

b *Simple effects treatment: Manipulation pen-mates and Ear biting at assessment II (P < 0.001)*.

**Table 5:** Frequency of behaviors (number of events/100 animals^*^10 min; LS-means ± standard error of means) for pigs in IP (improved pens) and CP (control pens) during assessments I (baseline) and II (middle of the fattening period) on Farm 2.

**Farm 2**	**Assessment I**		**Assessment II**		***P***		
	**CP**	**IP**	**CP**	**IP**	**T**	**A**	T^*^A
Manipulation objects	70.8 ± 19.9	66.5 ± 20.4	31.4 ± 9.8	36.3 ± 12.2	0.837	**0.004**	0.648
Manipulation straw/hay	–	105 ± 22.2	–	54.2 ± 11.8	–	**0.026**	–
Manipulation pen mates	48.6 ± 8.6	20.6 ± 6.9	86.3 ± 8.8	56.7 ± 8.8	**0.015**	**<0.001**	0.204
Tail biting^a^	14.3 ± 8.2	8.1 ± 6.8	55.5 ± 22.5	14.9 ± 5.5	**0.033**	0.067	0.478
Ear biting	23.3 ± 5.3	4.1 ± 2.8	45.2 ± 5.2	13.4 ± 3.5	**0.002**	**0.011**	0.446
Agonistic behavior	38.4 ± 8.3	10.3 ± 5.3	14.4 ± 3.1	8.3 ± 2.9	**0.005**	0.132	0.336

P-values are given for the effects of treatment, assessment and the interaction term (T^*^A) and are considered significant at P < 0.039 (bold values). –, effect not included in the model.

a *Absence of enrichment object was significant and thus retained in the model*.

**Table 6:** Incidence of behaviors (number of events/100 animals^*^10 min; median M, lower quartile Q1 and upper quartile Q3) for pigs in IP (improved pens) and CP (control pens) during assessments I (baseline) and II (middle of the fattening period) on Farm 3.

**Farm 3**	**Assessment I**						***P***	**Assessment II**						***P***
	**CP**			**IP**			**T**	**CP**			**IP**			**T**
	**M**	**Q1**	**Q3**	**M**	**Q1**	**Q3**		**M**	**Q1**	**Q3**	**M**	**Q1**	**Q3**	
Manipulation objects	156	119	192	152	80.0	200	0.773	23.6	17.4	73.6	38.3	8.3	70.0	0.773
Manipulation straw/hay	–	–	–	208	113	265	–	–	–	–	50.0	28.3	138	–
Manipulation pen mates	60.4	22.2	87.5	18.3	8.3	60.0	0.561	87.5	25.0	140	33.3	16.7	66.7	0.307
Tail biting	6.3	0.0	12.5	8.3	0.0	16.7	0.533	11.1	0.0	23.6	0.0	0.0	8.3	0.321
Ear biting	25.0	6.3	52.1	0.0	0.0	10.0	0.166	22.2	5.6	35.4	8.3	0.0	18.3	0.375
Agonistic behavior	37.5	24.3	79.9	40.0	28.3	95.0	0.381	59.0	34.0	64.6	0.0	0.0	10.0	**0.038**

*P-values are given for the treatment effect based on Mann-Whitney-U test (T) and are considered significant at P <0.048 (bold values). –, treatment effect not tested*.

On Farm 1, IP pigs manipulated straw numerically more frequently than the standard enrichment object. At the second assessment manipulation of pen-mates (*P*_*interaction*_ = 0.021) and ear biting (*P*_*interaction*_ = 0.002) was more frequently observed in CP than in IP pigs, while such differences were absent at assessment I. Tail biting did not differ between treatments. More agonistic interactions were observed in CP than in IP (*P* = 0.007).

Manipulation of the enrichment object did not differ between treatment groups on Farm 2. Manipulation of pen-mates occurred more frequently in CP than in IP (*P* = 0.015). Similarly, the number of tail and ear biting incidences as well as agonistic interactions was higher in CP than in IP (*P* = 0.033, *P* = 0.002 and *P* = 0.005, respectively).

On Farm 3, agonistic interactions were the only behavioral measure which differed between treatments during the second assessment, with less interactions occurring in IP pens (*P* = 0.038).

No treatment differences were found for any of the lesions or soiling of pigs on all three farms (Tables 7–9).

**Table 7:** Prevalence of lesions and soiled pigs (mean percentage of animals per pen; LS-means ± standard error of means) for pigs in IP (improved pens) and CP (control pens) during assessments I (baseline) and II (middle of the fattening period) on Farm 1.

**Farm 1**	**Assessment I**		**Assessment II**		**p**		
	**CP**	**IP**	**CP**	**IP**	**T**	**A**	T^*^A
Dirty animals	0.0^a^ ± 0.0	0.8^a^ ± 3.5	21.0^a^ ± 21.1	22.5^a^ ± 18.8	0.418	**<0.001**	–
Round lesions	4.5 ± 1.3	4.3 ± 1.4	6.6 ± 1.6	8.0 ± 2.0	0.722	**0.012**	0.536
Scratches	68.5 ± 6.2	66.7 ± 6.3	44.2 ± 3.9	44.7 ± 4.1	0.909	**<0.001**	0.684
Ear tip lesions	9.20 ± 8.3	7.7 ± 7.0	3.6 ± 3.3	2.5 ± 2.3	0.216	**<0.001**	0.56
Ear edge lesions	1.8 ± 0.6	0.5 ± 0.4	0.7 ± 0.5	0.2 ± 0.3	0.150	0.363	0.902
Tail lesions	2.6 ± 1.2	3.7 ± 1.7	1.8 ± 0.9	2.9 ± 1.4	0.213	0.323	0.818

P-values are given for the effects of treatment, assessment and the interaction term (T^*^A) and are considered significant at P < 0.037 (bold values).

a *Absence of enrichment object was significant and thus retained in the model*.

*^b^Interaction did not converge*.

**Table 8:** Prevalence of lesions and soiled pigs (mean percentage of animals per pen; LS-means LSM and standard error of means SEM) for pigs in IP (improved pens) and CP (control pens) during assessments I (baseline) and II (middle of the fattening period) on Farm 2.

**Farm 2**	**Assessment I**		**Assessment II**		***P***		
	**CP**	**IP**	**CP**	**IP**	**T**	**A**	T^*^A
Dirty animals	18.2 ± 5.3	10.2 ± 4.0	15.0 ± 3.7	11.5 ± 3.2	0.070	0.869	0.516
Round lesions	6.4 ± 2.5	5.2 ± 2.3	6.4 ± 2.5	7.5 ± 3.1	0.910	0.509	0.509
Scratches	54.3 ± 6.4	54.0 ± 6.9	35.4 ± 4.9	37.6 ± 5.7	0.796	**<0.001**	0.736
Ear tip lesions	3.3 ± 1.2	0.6 ± 0.6	6.5 ± 1.7	0.6 ± 0.6	0.060	0.276	0.281
Ear edge lesions	1.8 ± 1.0	2.6 ± 1.4	1.4 ± 0.7	0.0 ± 0.0	0.989	0.988	0.988
Tail lesions	3.8 ± 1.4	6.5 ± 2.4	3.4 ± 1.4	2.2 ± 1.3	0.888	0.121	0.200

P-values are given for the effects of treatment, assessment and the interaction term (T^*^A) and are considered significant at P < 0.039 (bold values).

**Table 9:** Prevalence of lesions and soiled pigs (median M, lower quartile Q1 and upper quartile Q3) for pigs in IP (improved pens) and CP (control pens) during assessments I (baseline) and II (middle of the fattening period) on Farm 3.

**Farm 3**	**Assessment I**						**P**	**Assessment II**						**P**
	**CP**			**IP**			**T**	**CP**			**IP**			**T**
	**M**	**Q1**	**Q3**	**M**	**Q1**	**Q3**		**M**	**Q1**	**Q3**	**M**	**Q1**	**Q3**	
Dirty animals	0.0	0.0	11.1	10.0	0.0	20.0	0.283	29.2	22.2	44.4	35.0	0.0	50.0	1.000
Round lesions	0.0	0.0	12.5	0.0	0.0	0.0	0.140	5.6	0.0	12.5	0.0	0.0	16.7	1.000
Scratches	41.0	37.5	75.0	50.0	20.0	66.7	0.936	34.7	22.2	50.6	50.0	16.7	66.7	0.575
Ear tip lesions	0.0	0.0	0.0	0.0	0.0	0.0	0.317	0.0	0.0	0.0	0.0	0.0	0.0	0.317
Ear edge lesions	0.0	0.0	0.0	0.0	0.0	0.0	0.317	0.0	0.0	0.0	0.0	0.0	0.0	1.000
Tail lesions	0.0	0.0	0.0	8.3	0.0	0.0	0.059	0.0	0.0	12.5	0.0	0.0	0.0	0.128

*P-values are given for the treatment effect (T) based on Mann-Whitney-U test and are considered significant at P <0.048 (bold values)*.

On Farm 1, ADG was higher by 71 g in IP than in CP (*P* = 0.003) whereas lean mean content did not differ between treatment groups (Table 10). On Farm 3, lean meat content was lower in IP (58.6%) than in CP (60.0%; *P* = *0.012)*. No differences in ADG or lean meat content were found on Farm 2.

**Table 10:** Performance data for control (CP) and improved (IP) pens.

	**Farm 1**			**Farm 2**			**Farm 3**		
	**CP**	**IP**	***P***	**CP**	**IP**	***P***	**CP**	**IP**	***P***
ADG (g)	828 ± 22	896 ± 20	**0.003**	736 ± 22	760 ± 22	0.178	714 ± 22	757 ± 22	0.241
n	16	21		12	12		6	6	
LMC (%)	60.9 ± 0.33	60.8 ± 0.31	0.793	60.6 ± 0.17	60.7 ± 0.19	0.914	60.0 ± 0.50	58.6 ± 0.57	**0.012**
n	345	306		215	155		49	31	

*n, number of feedings valves (ADG) or individual animals (LMC); LSM, least square means; SEM, standard error of means; ADG, average daily weight gain [g]; LMC, lean meat content [%]; T, treatment effect; P-values were considered significant for P < 0.037 (Farm 1), P <0.037 (Farm 2) and P <0.048 Farm 3 (bold values)*.

Animals on Farm 1 were treated for *Streptococcus spp*. (37% CP, 38% IP) and due to leg problems (3% CP, 2.8% IP). Pigs on Farm 2 received medication due to respiratory diseases (0.4% CP, 2.1% IP) and 3.9% of the pigs in CP on Farm 3 were treated following tail biting. Mortality rates were 1.6% (CP) and 1.6% (IP), 0.4% (CP) and 1.5% (IP) and 0% (CP) and 3% (IP) on Farm 1, Farm 2 and Farm 3, respectively.

## Discussion

The multi-farm approach of the present study enabled an intra-farm comparison of the interventions on three farms which used the most common husbandry system for pig fattening in Austria. A major challenge arose with respect to fitting a uniform study design to the three different farms (28). Most importantly, omission of tail docking was not possible to implement as an additional measure in the IP treatment on all three farms. As increased space allowance was achieved by reducing the number of animals per pen, the effect of a smaller group size in IP cannot be disentangled from lower stocking density. This combination of effects was inherent in the study design and would also be the case if this measure was implemented in practice, in particular by farmers, who are not able to implement constructional changes. Also, farms differed regarding group size, but overall group sizes can still be considered as small in a commercial farm setting in both, IP and CP (29). Furthermore, the number of pens per treatment and replications in Farm 3 was too small to apply the same hierarchical model as for the analysis of the other farms (26). Thus, data for Farm 3 was analyzed for each assessment separately using Mann-Whitney U-Test.

While we are aware of the limitations regarding the possibility to control for all risk factors and the differences between the farms, we also want to stress the strength of our multi-farm approach. Other studies have investigated similar questions under more controlled conditions e.g., on one experimental farm and/or investigating only effects of enrichment materials (9, 14, 30) and generated important scientific insights. However, by implementing the combination of measures on three different farms, we were able to investigate the potential for welfare improvement under real farm conditions and show the variability of effects, which may not only lead to more acceptance by farmers, but also increase the external validity of results.

The time slot chosen for assessment I seemed well suited for assessing indicators of physical appearance whereas it was a less adequate baseline for behavioral observations. Pigs on Farm 1 showed generally low activity during assessment I following transportation stress in contrast to Farm 2 and Farm 3, in which piglets had only been moved to another room or building when transferring them to the fattening pens. However, due to practical reasons and management conditions on the individual farms it was necessary to perform both observations for assessment I at the very first day of the fattening period. Thus, we refrained from discussing any effect of time on behavior.

In IP pens, manipulation of straw or hay was consistently higher than manipulation of the enrichment object, which is in line with studies on the properties of substrates for pigs emphasizing straw as the most suitable material (8, 9). Furthermore, straw was refilled on a regular basis and thus the novelty of the material may have been advantageous (31, 32). Positioning the racks above the trough was effective to prevent straw from quickly disappearing through the slats, which reduces availability of straw for the pigs and increases the risk of clogging the slurry system (33).

With the exception of tail biting on Farm 1, all pen-mate directed manipulative behaviors on Farm 1 and Farm 2 were significantly lower in IP than in CP whereas no differences were found on Farm 3 (for which statistical power was limited due to the lower number of pens investigated). Although some studies suggest that pigs with intact tails have a higher risk for tail biting outbreaks (4, 34, 35), tail biting occurrence did not differ or was even lower in IP irrespective of whether pigs had intact or docked tails. This suggests that, in accordance with e.g., (30, 36), the combination of increased space allowance and provision of straw in racks successfully contributed to the reduction of tail and ear biting and other pen-mate directed manipulative behaviors and therefore outweighed the higher risk by keeping tails intact (30). Furthermore, due to the lower number of pigs per pen, the animal-per-feeder ratio decreased, which additionally may have contributed to a decrease in tail biting behavior in IP. If feeder-space allows pigs to feed simultaneously this may prevent tail biting resulting from attacks from the rear at the trough during feeding (6, 37). Therefore, our findings may only apply if stocking density is reduced without reducing feeder space at the same time.

Contrasting the findings of the behavioral observations but in accordance with other studies [e.g., (14, 38)], there were no treatment effects for any of the clinical indicators. This leads to the conclusion that damaging behavior may gain physical relevance at higher occurrence only (32). Furthermore, it has to be emphasized that prevalence of tail lesions was generally low (ranging from 0 to 8.3%, including superficial scratches), irrespective of whether tails were intact or docked, compared to the prevalence found in other studies e.g., 1.5–2.8% (39), 7.6% (12), 5–23% (14), 16.3% (40), 0–23% (35), 24.1% (32) and 54% (41). The absence of a significant effect on tail lesions while there was at least numerically less tail biting in IP (significant for Farm 2), may be due to a floor effect. On the one hand, a low prevalence already under control conditions, e.g., due to risk assessment prior to the study and good management on the participating farms, may be difficult to be further reduced. On the other hand, live on-farm observations may underestimate the true prevalence of tail lesions compared to abattoir inspections after scalding and dehairing (42) and especially in undocked pigs tail length should be additionally considered when assessing tail lesion status (43).

Round skin lesions often result from pen-mate directed behavior including belly-nosing (44). Although a lower incidence of pen-mate directed behavior in IP suggests fewer round lesions in the IP pigs, no treatment effect was found. Similarly, reduced agonistic behavior in IP was not reflected in a lower prevalence of related body skin lesions (45). It is important to point out that providing one rack per max. 13 pigs apparently did not provoke additional fights (or signs thereof) over access to enrichment material. An earlier study has shown that even at a higher animal:rack ratio the incidence of displacements was low (33).

In contrast to the concerns of farmers, that lower stocking densities would lead to increased soiling of the animals, as the dung would not sufficiently be pushed through the slats, cleanliness of animals in the two treatments did not differ. Although cleanliness of the pens themselves was not assessed, it can be assumed that either the manure sufficiently disappeared through the slats due to animal activity or that due to the lower stocking density pigs were able to create a separate dunging area so that the lying area remained clean (46). In addition to space allowance, pen soiling is influenced by other factors, such as pen structuring, floor type or ambient temperature (47), which were similar at farm level for IP and CP pens. These other factors may have outweighed possible effects of differences in stocking density.

Pigs in IP on Farm 1 had a significantly higher ADG whereas no difference was found between treatments on Farm 2 and Farm 3. Comparing similar space allowances (0.8 m^2^ vs. 1.0 m^2^ per pig), Vermeer et al. (48) also found significantly higher ADG for pigs in pens with higher space allowance. Other studies investigating the combination of higher space allowance and environmental enrichment in fattening (49) or weaner pigs (50) also conclude that these measures improved growth rates. It has been hypothesized that the effect is due to a lower stress level in the animals enhancing the exploitation of genetic growth potential (51) e.g., by better access to food and water. Maybe also the lower animal:feeder ratio contributed to an improved fattening performance as all animals were able to feed simultaneously without difficulties. Interestingly, ADG on Farm 1 was generally higher than on the other two farms. More space and straw/hay racks as environmental enrichment may therefore lead to higher ADG on better performing farms while this effect may be superimposed by other factors such as feeding regime (e.g., absence of multi-phase feeding) or infections [e.g., endoparasites (52)].

Lean meat percentage was similar on all three farms and was only significantly lower in pigs in IP on Farm 3. A similar observation was made by Beattie et al. (49), who found that pigs kept in enriched environments had increased levels of backfat. They argued that pigs may have started to deposit more fat due to reaching their maximum potential for lean deposition faster than pigs in the barren environment.

Treatment incidences and mortality rates of CP and IP pigs did not show any consistent pattern. Infection with *Streptococcus* spp. may be attributed to tail biting wounds, but also any other lesion such as skin lesions may be the portal of entry for these pathogens. Thus, linking farmers' treatment records to other outcomes of this study related to treatment differences did not provide any conclusive results.

The results of our study showed that the implementation of reduced stocking density and straw/hay racks as additional environmental enrichment were able to improve animal welfare to some extent. However, reducing stocking density will reduce farm revenues by reducing the number of pigs sold and the use of straw/hay racks may increase costs for equipment and labor. Furthermore, this will entail effects for the whole production chain. Due to the lower number of fattening pigs also a lower number of sows will be needed. Finally, the demand for pork products still has to be met, which may not be the case if less pigs are fattened on farms. Therefore, before implementing these measures on a larger scale, effects on the whole supply chain have to be considered to avoid negative economic consequences.

## Conclusions

The results of the present study suggest that reduced stocking density (resulting in a space allowance of 1.0 m^2^ compared to 0.7 m^2^ per animal) and provision of straw or hay in a rack improve welfare of fattening pigs under commercial housing conditions. Positive effects were observed in particular with respect to an increase in exploratory behavior using enrichment materials rather than being redirected toward pen-mates. Therefore, a straw rack may present a suitable compromise regarding appropriate enrichment material for pigs and practicality in slurry systems. Data from Farm 2 also suggest that under these circumstances an omission of tail docking might be possible in fully slatted housing systems without increasing the risk for tail biting. Although the interventions may only mean a limited improvement of the situation for fattening pigs kept in intensive housing conditions, a wide implementation could immediately improve welfare for a large number of animals given that economic disadvantages for farmers are compensated.

## Data Availability Statement

The raw data supporting the conclusions of this article will be made available by the authors, without undue reservation.

## Ethics Statement

At the time the study was conducted (2013–2014), no Ethics Committee was available at the University of Natural Resources and Life Sciences, Vienna. The work did not require experimental licensing because it did not involve any invasive procedures. Furthermore, farms were in compliance with EU and national legislation on pig welfare. Written informed consent was obtained from the owners for the participation of their animals in this study.

## Author Contributions

KS drafted the manuscript, collected data on farms and abattoirs, performed statistical analyses, and contributed to the study design and farm acquisition. LW collected and analyzed behavioral data. CW and CL acquired funding of the study, directed study design, and substantially contributed to statistical analyses and writing of the manuscript. All authors contributed to the article and approved the submitted version.

## Funding

BILLA AG was gratefully acknowledged for funding this study, which was conducted in the course of the Doctoral School of Sustainable Development (dokNE II) at the University of Natural Resources and Life Sciences, Vienna.

## Conflict of Interest

The authors declare that the research was conducted in the absence of any commercial or financial relationships that could be construed as a potential conflict of interest.

## Publisher's Note

All claims expressed in this article are solely those of the authors and do not necessarily represent those of their affiliated organizations, or those of the publisher, the editors and the reviewers. Any product that may be evaluated in this article, or claim that may be made by its manufacturer, is not guaranteed or endorsed by the publisher.

## References

[1] Jensen T Kold Nielsen C Vinther J D'Eath RB. The effect of space allowance for finishing pigs on productivity and pen hygiene. Livest Sci. (2012) 149:33–40. 10.1016/j.livsci.2012.06.018

[2] Turner SP Ewen M Rooke JA Edwards SA. The effect of space allowance on performance, aggression and immune competence of growing pigs housed on straw deep-litter at different group sizes. Livest Prod Sci. (2000) 66:47–55. 10.1016/S0301-6226(00)00159-7

[3] Vermeer HM de Greef KH Houwers HWJ. Space allowance and pen size affect welfare indicators and performance of growing pigs under Comfort Class conditions. Livest Sci. (2014) 159:79–86. 10.1016/j.livsci.2013.10.021

[4] Schrøder-Petersen DL Simonsen HB. Tail biting in pigs. Vet J. (2001) 162:196–210. 10.1053/tvjl.2001.0605 11681870

[5] Valros A Ahlström S Rintala H Häkkinen T Saloniemi H. The prevalence of tail damage in slaughter pigs in Finland and associations to carcass condemnations. Acta Agric Scand - Sect A Anim Sci. (2004) 54:213–9. 10.1080/09064700510009234

[6] EFSA. The risks associated with tail biting in pigs and possible means to reduce the need for tail docking considering the different housing and husbandry systems. In: Scientific Opinion of the Panel on Animal Health and Welfare. (2007).

[7] van de Weerd H Ison S. Providing effective environmental enrichment to pigs: how far have we come? Animals. (2019) 9:254. 10.3390/ani9050254 31117191 PMC6562439

[8] van de Weerd HA Day JEL. A review of environmental enrichment for pigs housed in intensive housing systems. Appl Anim Behav Sci. (2009) 116:1–20. 10.1016/j.applanim.2008.08.001

[9] Chou J-Y Drique C Sandercock D D'Eath R O'Driscoll K. Rearing undocked pigs on fully slatted floors using multiple types and variations of enrichment. Animals. (2019) 9:139. 10.3390/ani9040139 30986987 PMC6523089

[10] Wallgren T Lundeheim N Wallenbeck A Westin R Gunnarsson S. Rearing pigs with intact tails—experiences and practical solutions in Sweden. Animals. (2019) 9:812. 10.3390/ani9100812 31619014 PMC6826450

[11] BMGF. Verordnung der Bundesministerin für Gesundheit und Frauen über die Mindestanforderungen für die Haltung von Pferden und Pferdeartigen, Schweinen, Rindern, Schafen, Ziegen, Schalenwild, Lamas, Kaninchen, Hausgeflügel, Straußen und Nutzfischen (1). Tierhaltu: BMGF (2004).

[12] Van Staaveren N Calderón Díaz JA Garcia Manzanilla E Hanlon A Boyle LA. Prevalence of welfare outcomes in the weaner and finisher stages of the production cycle on 31 Irish pig farms. Ir Vet J. (2018) 71:9. 10.1186/s13620-018-0121-5 29599967 PMC5869776

[13] Grümpel A Krieter J Veit C Dippel S. Factors influencing the risk for tail lesions in weaner pigs (*Sus scrofa*). Livest Sci. (2018) 216:219–26. 10.1016/j.livsci.2018.09.001

[14] Brandt P Hakansson F Jensen T Nielsen MBF Lahrmann HP Hansen CF Forkman B. Effect of pen design on tail biting and tail-directed behaviour of finishing pigs with intact tails. Animal. (2020) 14:1034–42. 10.1017/S1751731119002805 31735187

[15] Council of the European Union. Council Directive 2008/120/EC of 18 December 2008 laying down minimum standards for the protection of pigs as regards measures to reduce the need for tail-docking (Codified version). Off. J. Eur. Union (2009).

[16] Zonderland JJ Schepers F Bracke MBM Den Hartog LA Kemp B Spoolder HAM. Characteristics of biter and victim piglets apparent before a tail-biting outbreak. Animal. (2011) 5:767–75. 10.1017/S1751731110002326 22439999

[17] Zwicker B Gygax L Wechsler B Weber R. Short- and long-term effects of eight enrichment materials on the behaviour of finishing pigs fed ad libitum or restrictively. Appl Anim Behav Sci. (2013) 144:31–8. 10.1016/j.applanim.2012.11.007

[18] Statham P Green L Bichard M Mendl M. Predicting tail-biting from behaviour of pigs prior to outbreaks. Appl Anim Behav Sci. (2009) 121:157–64. 10.1016/j.applanim.2009.09.011

[19] Beattie VE Breuer K O'Connell NE Sneddon IA Mercer JT Rance KA. Factors identifying pigs predisposed to tail biting. Anim Sci. (2005) 80:307–12. 10.1079/ASC40040307

[20] Schrøder-petersen* DL Simonsen HB Lawson LG. Tail-in-mouth behaviour among weaner pigs in relation to age, gender and group composition regarding gender. Acta Agric Scand Sect A Anim Sci. (2003) 53:29–34. 10.1080/09064700310002017

[21] Camerlink I Turner SP. The pig's nose and its role in dominance relationships and harmful behaviour. Appl Anim Behav Sci. (2013) 145:84–91. 10.1016/j.applanim.2013.02.008

[22] Canali E Keeling L. Welfare Quality® project: from scientific research to on farm assessment of animal welfare. Ital J Anim Sci. (2009) 8:900–3. 10.4081/ijas.2009.s2.900

[23] Weissenbacher-Lang C Voglmayr T Waxenecker F Hofstetter U Weissenböck H Hoelzle K. Porcine ear necrosis syndrome: a preliminary investigation of putative infectious agents in piglets and mycotoxins in feed. Vet J. (2012) 194:392–7. 10.1016/j.tvjl.2012.05.026 22784419

[24] Goossens X Sobry L Ödberg F Tuyttens F Maes D De Smet S. Population-based on-farm evaluation protocol for comparing the welfare of pigs between farms. Anim Welf. (2008) 17:35–41.

[25] SAS Institute Inc,. Base SAS ® 9.4 Procedures Guide High-Performance Procedures Sixth Edition SAS ® Documentation. (2016). Available online at: https://go.documentation.sas.com/api/collections/pgmsascdc/9.4_3.5/docsets/procstat/content/procstat.pdf?locale=en#nameddest=titlepage (accessed November 23, 2021).

[26] Moineddin R Matheson FI Glazier RH. A simulation study of sample size for multilevel logistic regression models. BMC Med Res Methodol. (2007) 7:34. 10.1186/1471-2288-7-34 17634107 PMC1955447

[27] Benjamini Y Hochberg Y. Controlling the false discovery rate: a practical and powerful approach to multiple testing. J R Stat Soc Ser B. (1995) 57:289–300. 10.1111/j.2517-6161.1995.tb02031.x

[28] Schodl K Leeb C Winckler C. Developing science–industry collaborations into a transdisciplinary process: a case study on improving sustainability of pork production. Sustain Sci. (2015) 10:639–51. 10.1007/s11625-015-0329-1

[29] Verdon M Rault JL. Aggression in group housed sows and fattening pigs. In: Špinka M. Camerlink I. (eds) Advances in Pig Welfare (New York, NY: Elsevier Inc.), 235–260. 10.1016/B978-0-08-101012-9.00006-X

[30] Larsen MLV Andersen HML Pedersen LJ. Which is the most preventive measure against tail damage in finisher pigs: tail docking, straw provision or lowered stocking density? Animal. (2018) 12:1260–7. 10.1017/S175173111700249X 29094665

[31] Trickett SL Guy JH Edwards SA. The role of novelty in environmental enrichment for the weaned pig. Appl Anim Behav Sci. (2009) 116:45–51. 10.1016/j.applanim.2008.07.007

[32] Chou J-Y Sandercock DA D'Eath RB O'Driscoll KA. High enrichment replenishment rate reduces damaging behaviors and increases growth rate in undocked pigs kept in fully slatted pens. Front Vet Sci. (2020) 7:889. 10.3389/fvets.2020.584706 33282931 PMC7691579

[33] Zwicker B Gygax L Wechsler B Weber R. Influence of the accessibility of straw in racks on exploratory behaviour in finishing pigs. Livest Sci. (2012) 148:67–73. 10.1016/j.livsci.2012.05.008

[34] Nannoni E Valsami T Sardi L Martelli G. Tail docking in pigs: a review on its short- and long-term consequences and effectiveness in preventing tail biting. Ital J Anim Sci. (2014) 13:3095. 10.4081/ijas.2014.3095

[35] Lahrmann HP Busch ME D'Eath RB Forkman B Hansen CF. More tail lesions among undocked than tail docked pigs in a conventional herd. Animal. (2017) 11: 1825–31. 10.1017/S1751731117000490 28294097

[36] Beattie VE Walker N Sneddon IA. An investigation of the effect of environmental enrichment and space allowance on the behaviour and production of growing pigs. Appl Anim Behav Sci. (1996) 48:151–8. 10.1016/0168-1591(96)01031-3

[37] D'Eath RB Arnott G Turner SP Jensen T Lahrmann HP Busch ME. Injurious tail biting in pigs: how can it be controlled in existing systems without tail docking? Animal. (2014) 8:1479–97. 10.1017/S1751731114001359 25130712

[38] van Staaveren N Hanlon A Boyle LA. Damaging behaviour and associated lesions in relation to types of enrichment for finisher pigs on commercial farms. Animal. (2019) 9:ani9090677. 10.3390/ani9090677 31547306 PMC6770820

[39] Ursinus WW Van Reenen CG Kemp B Bolhuis JE. Tail biting behaviour and tail damage in pigs and the relationship with general behaviour: Predicting the inevitable? Appl Anim Behav Sci. (2014) 156:22–36. 10.1016/j.applanim.2014.04.001

[40] Kritas S Morrison R. An observational study on tail biting in commercial grower-finisher barns. J Swine Heal Prod. (2004) 12:17–22. Available online at: https://www.aasv.org/shap/issues/v12n1/v12n1p17.pdf (accessed November 23, 2021).

[41] Telkänranta H Bracke MBM Valros A. Fresh wood reduces tail and ear biting and increases exploratory behaviour in finishing pigs. Appl Anim Behav Sci. (2014) 161:51–9. 10.1016/j.applanim.2014.09.007

[42] Carroll GA Boyle LA Teixeira DL van Staaveren N Hanlon A O'Connell NE. Effects of scalding and dehairing of pig carcasses at abattoirs on the visibility of welfare-related lesions. Animal. (2016) 10:460–7. 10.1017/S1751731115002037 26412112

[43] Valros A Välimäki E Nordgren H Vugts J Fàbrega E Heinonen M. Intact tails as a welfare indicator in finishing pigs? scoring of tail lesions and defining intact tails in undocked pigs at the abattoir. Front Vet Sci. (2020) 7:405. 10.3389/fvets.2020.00405 32766295 PMC7378396

[44] Main RG Dritz SS Tokach MD Goodband RD Nelssen JL Loughin TM. Effects of weaning age on postweaning belly-nosing behavior and umbilical lesions in a multi-site production system. J Swine Heal Prod. (2005) 13:259–64. Available online at: https://www.aasv.org/shap/issues/v13n5/v13n5p259.pdf (accessed November 23, 2021).

[45] Turner SP Farnworth MJ White IMS Brotherstone S Mendl M Knap P. The accumulation of skin lesions and their use as a predictor of individual aggressiveness in pigs. Appl Anim Behav Sci. (2006) 96:245–59. 10.1016/j.applanim.2005.06.009

[46] Fu L Li H Liang T Zhou B Chu Q Schinckel AP. Stocking density affects welfare indicators of growing pigs of different group sizes after regrouping. Appl Anim Behav Sci. (2016) 174:42–50. 10.1016/j.applanim.2015.10.002

[47] Nannoni E Aarnink AJA Vermeer HM Reimert I Fels M Bracke MBM. Soiling of pig pens: a review of eliminative behaviour. Animals. (2020) 10:2025. 10.3390/ani10112025 33153115 PMC7693532

[48] Vermeer HM Dirx-Kuijken NCPMM Bracke MBM. Exploration feeding and higher space allocation improve welfare of growing-finishing pigs. Animals. (2017) 7:36. 10.3390/ani7050036 28468261 PMC5447918

[49] Beattie VE O'Connell NE Moss BW. Influence of environmental enrichment on the behaviour, performance and meat quality of domestic pigs. Livest Prod Sci. (2000) 65:71–9. 10.1016/S0301-6226(99)00179-7

[50] Munsterhjelm C Peltoniemi OAT Heinonen M Hälli O Karhapää M Valros A. Experience of moderate bedding affects behaviour of growing pigs. Appl Anim Behav Sci. (2009) 118:42–53. 10.1016/j.applanim.2009.01.007

[51] Hyun Y Ellis M Riskowski G Johnson RW. Growth performance of pigs subjected to multiple concurrent environmental stressors. J Anim Sci. (1998) 76:721. 10.2527/1998.763721x 9535330

[52] Vlaminck J Düsseldorf S Heres L Geldhof P. Serological examination of fattening pigs reveals associations between *Ascaris suum*, lung pathogens and technical performance parameters. Vet Parasitol. (2015) 210:151–8. 10.1016/j.vetpar.2015.04.012 25952722

